# Critical Issues in the Management of Agitation, Aggression, and End-of-Life in Delusional Disorder: A Mini-Review

**DOI:** 10.3390/healthcare11040458

**Published:** 2023-02-05

**Authors:** Alexandre González-Rodríguez, Mary V. Seeman, Eloïsa Román, Mentxu Natividad, Carmen Pagés, Camila Ghigliazza, Laura Ros, José A. Monreal

**Affiliations:** 1 Department of Mental Health, Mutua Terrassa University Hospital, University of Barcelona (UB), 5 Dr. Robert Square, 08221 Terrassa, Spain; 2 Department of Psychiatry, University of Toronto, #605, 260 Heath Street West, Toronto, ON M5T 1R8, Canada; 3 Institut de Neurociències, UAB, CIBERSAM, 08221 Terrassa, Spain

**Keywords:** delusional disorder, psychosis, old age, agitation, end-of-life

## Abstract

*Background***:** Compared to other psychotic disorders, there is little information about staging care in delusional disorder (DD). Unlike schizophrenia, this is a disorder that begins in middle age, a time at which chronic medical comorbidities have already begun to impact global functioning. With age, the combination of psychological and somatic conditions leads to new behaviours, e.g., agitation, aggression, and behaviours that require specific preventive and interventive measures. With further age, knowledgeable end-of-life care becomes necessary for this population. *Aim*: The aim of this article was to review existing evidence on the management of these successive phases. *Methods*: We conducted a narrative review using PubMed and ClinicalTrials.gov and searched for the following terms: (agitation OR aggressivity OR aggression OR palliative OR “end-of-life”) AND (“delusional disorder”). *Results*: We found that the literature was sparse. Existing evidence suggests that medical causes are frequently at the root of agitation and aggression. With respect to management, de-escalation strategies are generally preferred over pharmacotherapy. Specific delusional syndromes, e.g., de Clérambault, Othello, Capgras, Fregoli, as well as folie à deux, are associated with aggression. The somatic subtype of DD is the one most often requiring palliative care at the end of life. *Conclusions*: We conclude that insufficient attention has been given to the care needs of the accelerated aging process in DD.

## 1. Introduction

Delusional disorder (DD) is a psychotic disorder related to schizophrenia but distinctive in several ways. It is characterised by the presence of monosymptomatic–systematised delusions that morbidly preoccupy the patient. Prominent affective symptoms, hallucinations, and thought disorders, as seen in other psychotic disorders, are usually absent [1]. This means that, outside the sphere of the generally single delusion, global functioning is relatively intact.

Although epidemiological data are sparse and contested, most available studies put the prevalence of DD at around 0.02% [1]. The onset usually occurs in the middle or late-middle years [2]. In one register study of 5222 patients aged 65 and older, stratified by age and sex, Copeland and colleagues found the prevalence of DD to be 0.04% (versus 0.12% for schizophrenia); the incidence was 15.6 per 100,000 per year [3]. The relative rarity of this disorder means that, compared to other psychoses, it has been little studied. Because medical comorbidity, brain changes, cognitive decline, and poor pharmacological response rapidly increase in DD as patients age [2], it is clinically important to shed more light on the progressive stages of this condition.

Traditionally, DD has been classified into seven subtypes according to the content of the primary delusion: persecutory, erotomanic, grandiose, jealous, somatic, mixed, and unspecified types [1]. Although this classification has been widely accepted, other categorizations have also been proposed. For instance, Wustmann and colleagues classify delusional content in DD into three main groups: erotocentric (including erotomania and jealousy), somatocentric (somatic and hypochondriacal delusions), and securocentric (persecutory, querulous, and delusions of reference [4]. As patients age, the persecutory type becomes the most prevalent, with somatic and jealous types following close behind [2,5]. There is a suggestion that the specificity of delusional themes may be associated with the location of brain lesions as visualised on brain imaging. Holt and Albert [5] found a correlation between delusions of persecution and frontotemporal lesions in the left hemisphere while delusional misidentification was associated with lesions in the right hemisphere.

As with schizophrenia, DD can be accompanied by additional psychiatric comorbidities and affective disorders. The rate of comorbid depressive symptoms in DD is approximately 31.9%. In one register study, 67.5% of patients were treated with antidepressants [6]. The suicide risk rate is reported to be from 8 to 21%, most frequent in patients with somatic and persecutory delusions [7,8]. Because of the relatively late onset age, chronic medical conditions are frequent comorbidities. The Andalusian Delusional Disorder case register study (DelirANda) of 1452 DD patients found serious medical conditions in 66% of registered patients [9]; type 2 diabetes was the most frequent medical comorbidity found. The prevalence of specific cancers has been reported as high in related psychotic disorders [10,11], but has not been studied in DD research. The need for end-of-life or palliative care in this disorder remains uncharted territory. Behavioural comorbidities, such as agitation and aggressive behaviours that result from age and cognitive decline, are known to occur in schizophrenia [12] but have not been well-investigated in the DD population.

In this review, we summarise the little evidence that exists on the staging of care for improved management of agitation, aggression, and end-of-life issues for DD patients as they age. Borrowing from the larger literature on the treatment of aging delusional patients with closely related diagnoses, we end with several recommendations.

## 2. Methods

### 2.1. Screening and Selection Process

We conducted a narrative review based on the following electronic searches: PubMed and ClinicalTrials.gov, for papers written in English, German, French, and Spanish prior to September 2022 referring in their titles or abstracts to agitation/aggression, aggressive behaviours, or end-of-life care in delusional disorders. Additionally, reference lists of studies we included were screened for research of potential interest to our aim e.g., information on the effective treatment of aging delusional patients with related diagnoses. 

To start with, we used the following search terms: (agitation OR aggressivity OR aggression OR palliative OR “end-of-life”) AND (“delusional disorder”). The screening and selection of papers were done by A.G.R., M.V.S., and E.R. A total of 41 studies were judged eligible.

### 2.2. Inclusion/Exclusion Criteria

Studies were initially included if participants were patients with DD diagnosed according to the Diagnostic and Statistical Manual for Mental Disorders (DSM) or the International Classification of Diseases (ICD), or else were patients described as showing persistent delusions. Randomised controlled trials, observational and prospective/retrospective studies, and case reports or case series were all included.

## 3. Results

### 3.1. Management of Psychomotor Agitation and Aggressive Behaviour in Delusional Disorder 

Psychomotor agitation refers to anxious restlessness, pacing, fidgeting, and handwringing, a commonly seen clinical feature of several psychiatric diseases. Agitation can result from associated pain, comorbid neurological conditions, such as encephalitis or delirium or dementia, comorbid thyroid disease, hypoxia, hyponatremia, hypocalcaemia, or hypoglycemia, and drug withdrawal. It can be antipsychotic-induced in the form of akathisia [13].

If not effectively addressed, agitation can escalate to unpredictable, violent, and dangerous behaviour [14,15]. Fortunately, many causes of agitation, once diagnosed, are medically reversible. Chronic anxiety/agitation caused by underlying neurological conditions, however, tends to increase with age and is difficult to manage. 

In patients diagnosed with psychosis, the literature recommends psychosocial interventions first and the use of pharmacological treatments or physical restraints only if de-escalation measures fail [15]. Psychosocial strategies consist of ensuring a safe and calm environment, establishing verbal contact, reassuring patients that they are not alone, and that family members or care providers are there to support them. Concise communication and frequent soothing repetition of assurance are recommended. A strong recommendation is to respect patients’ personal space and continuously assess and monitor safety risks. Active listening is considered vital, as is verbally and non-verbally expressed empathy for the patient’s predicament. Reframing is advised for situations that are particularly worrisome for patients. Other suggested interventions are to discuss patients’ behavioural options and explore alternatives to worry or hostility. A recent review of expert opinions on the management of agitation highlights the need to consider verbal de-escalation and environmental modification techniques as the first choice of treatment [16]. These are opinions based on a consensus extracted from 124 published studies. 

Identification and early treatment of all causes of agitation are vital, according to the literature, for the safety of the person and immediate others [16,17]. 

When deemed necessary, pharmacological measures for acute agitation include benzodiazepines and both first- and second-generation antipsychotics in low doses to calm patients without producing over-sedation. Oral or inhaled formulations are preferred over intramuscular injections, which, in turn, are preferred over intravenous administration. Decisions about the specific choice and dosing of benzodiazepine and antipsychotics depend on side-effect profiles, drug–drug interactions, and pharmacokinetic and pharmacodynamic characteristics appropriate to the population’s age. With increasing age, safety, and treatment response to medications rest on the presence of brain changes, cognitive defects, and renal health [3]. A complete physical and psychological assessment is required to determine the safest and most effective dose range of medication. 

Protocols for physical restraint underscore that it be restricted to situations where repeated attempts at de-escalation and pharmacological intervention have not worked, and the estimated risk of violence is high [18]. Restraint is a last resort because it can cause injuries. Reported injuries have included dehydration, choking, circulatory, skin problems, loss of strength and mobility, as well as negative psychological effects on patients and staff. As patients age, these adverse effects can become serious, sometimes leading to death [19]. Physical integrity and life are at stake in determining whether physical restraint is necessary, but human values, such as patient autonomy and dignity, also hang in the balance [20].

While escalation to aggression has been extensively investigated in the context of schizophrenia, and especially in the literature on emergency psychiatry [21], very few studies have addressed this issue specifically in DD. 

A case register-descriptive study that included 467 patients with DD at a community mental health service in South Barcelona, Spain, reported that ideas of reference, irritability, depressive mood, and aggressiveness (15%) were the most common symptoms and signs accompanying delusional ideas [22]. In this study, most participants exhibited persecutory, jealous, and mixed delusions. The investigators found that behaviour necessitating police intervention was associated with single or separated marital status, spousal absence being viewed as a cause, or perhaps a consequence, of spousal absence. The same research group carried out a cross-sectional study of 86 outpatients with DSM-IV DD [23]. They factor-analysed results of the Positive and Negative Syndrome Scale (PANSS) and found four consistent factors that were positively associated with clinical variables: paranoid, cognitive, schizoid, and affective factors. The paranoid dimension was correlated with paranoid personality disorders, poor adherence to treatment, and poor therapeutic response. Paranoid patients also had more legal problems than those with predominantly affective, cognitive, or schizoid symptoms, suggesting that the group of “pure” (more exclusively paranoid) patients were those most likely to show aggression.

Serretti and collaborators investigated differential symptomatology in a large sample of 1351 research participants with major psychoses [24], grouped into the following diagnostic categories: DD (*n* = 93), schizophrenia (*n* = 358), bipolar disorder (*n* = 511), and major depressive disorder (*n* = 389). In all patients, the Operational Criteria for Psychotic Illness checklist (OPCRIT) was used to assess symptoms. Psychomotor symptoms, most of which were related to depressive symptomatology, were more commonly found in schizophrenia than in DD. The investigators concluded that depressive symptoms were present in both populations, DD, and schizophrenia, but that depression was more severe in DD. In a cohort of patients with and without a history of suicide attempts, Craig and Bivens investigated the association between suicidality and personality disorders [25]. Patients with a history of suicide attempts had higher scores on both depression and paranoid personality disorders, suggesting that self-harm was associated with paranoid features. The association between aggression, suicidality, and DD has not, yet, been more fully investigated.

A recent cross-sectional study carried out by Nederlof and collaborators examined the tendency to overestimate threat or control by others, sometimes called threat/control-override symptoms. The researchers wondered whether this tendency or anger/anxiety plus positive symptoms of psychosis were likely to lead to aggressive acting out [26]. Patients with DD, schizophrenia, and schizoaffective disorders were evaluated. The findings were that it was the disposition to anger that was significantly associated with aggressive behaviour in all psychosis patients.

A recent retrospective study explored the association between DD and criminally violent behaviour in 346 patients assessed by the Overt Aggression Scale (OAS). The aim of the study was to identify predictors of activity judged as criminally offensive in that police intervention had been required [27]. Of the 346 DD study participants, 172 were categorised as offenders and 174 as non-offenders. Offenders were older than non-offenders. No statistically significant differences were found between the two groups in terms of age at disease onset or gender. Homicide and attempted homicide were most frequent in patients with jealous delusions; verbal assault and other aggressive crimes were most common in patients with persecutory delusions. Older age, high scores on the OAS scale, and persecutory delusions were associated with a higher risk of offense, suggesting that, with age, persecutory delusions tend to increasingly manifest in aggressive behaviour in patients with this condition.

Very few studies have recommended gold-standard interventions in curbing aggressive behaviour in DD. A retrospective study from the Clinical Hospital of Psychiatry and Neurology in Romania described the clinical features of 19,000 inpatients from 2010 to 2019 [28]. Five hundred and four patients received clozapine specifically for aggressivity; the sample included cases of DD (unfortunately only two), obsessive-compulsive disorder, and mood disorders. Clozapine was found to be efficacious and safe in patients with treatment-refractory aggressive behaviour. Clozapine, however, is not recommended in older age DD patients because of clozapine’s many adverse effects, especially low blood pressure and sedation [29].

In summary, evidence shows that agitation in patients with psychoses needs to be promptly and effectively treated in order to avoid physical violence and other aggressive behaviours that appear to increase with age. Medical causes need to be ascertained and treated. De-escalation strategies are preferred as immediate intervention, followed, if necessary, by pharmacological strategies that do not over-sedate the patient or cause untoward adverse effects. The literature agrees that physical restraints should be avoided whenever possible. While there is no formal guidance specific to patients with DD, because of age and potential neurological deficits, this is the population at high risk for adverse effects of both pharmaceuticals and physical restraint measures.

### 3.2. Classical Delusional Syndromes Associated with Aggression

Certain classical delusional syndromes have been specifically associated with aggression. The de Clérambault syndrome, the Othello syndrome, Folie à Deux, and its variants, have all received considerable attention in forensic services [30], as have Capgras and Fregoli syndromes [31]. 

De Clérambault syndrome is characterised by erotomanic delusions (the false belief that one has aroused the passionate love of a prominent person). In the early stages, this is a grandiose delusion that enhances a person’s self-esteem but, over time, as the imagined lover fails to respond in the hoped-for way, patients can become resentful and act out aggressively against the ‘lover’ or whoever stands in the way of the imagined relationship. While delusions of erotomania have been reported to occur more frequently in women than men, aggressive behaviour in the context of this syndrome is more common in men [32]. Seeman has proposed a staged treatment plan for erotomania [33]. The suggestion is to first establish a therapeutic alliance with a focus on understanding the psychological factors at the root of the delusion and the psychosocial factors contributing to the maintenance of the delusion. The next stage consists of the provision of social support and psychotherapeutic strategies aimed at the restoration of self-esteem. The third stage in this approach is the gradual introduction of techniques to correct cognitive biases. Medication (antipsychotics) and environmental risk management are important to overall management once a therapeutic alliance is established. There is no objective evidence for the effectiveness of this proposed approach, however.

The Othello syndrome is another name for the jealousy subtype of DD [34]. Several studies have supported pharmacological (antipsychotic) treatment combined with dialectical behavioural therapy as the treatment of choice. Seeman has emphasised the interpersonal aspect of the delusion of jealousy [35]. This delusion can be triggered by the provocative behaviour of the partner and maintained by reasoning biases and by the benefits (for both partners) that it initially bestows on the relationship (increases in sexual passion, high emotionality, gifts, declarations of loyalty). In the long run, however, delusional jealousy poses dangerous risks to the patient, the partner, and the imagined rival(s). Treatment recommendations include couple therapy, a strong cognitive focus, antipsychotic medication, and psychotherapeutic interventions that enhance the self-esteem of both partners and address the solidarity of the existing relationship. Whereas treatment effectiveness lacks a firm evidence base, the assessment of aggressive risk factors and attention to safety is generally acknowledged as mandatory. 

Another clinical syndrome linked to DD is shared psychotic disorder or folie à deux [36]. Some reports warn that two or more people sharing a common delusion can spur each other to violence, and fatalities have been reported. Treatment usually involves the separation of delusional partners and the use of antipsychotic medications. A classic paper revealed that males and females and younger and older patients suffered from folie à deux with equal frequency; 90% consisted of married couples or two siblings or parent-child dyads [37]. Comorbid dementia, depression, and mental retardation were commonly present, as were auditory hallucinations. Social isolation characterised the circumstances of most folie à deux dyads. Available evidence supports a primary delusional, dominant patient who ‘infects’ a subordinate person. Reif and Pfuhlman contend that, for this to occur, both partners must have an underlying genetic propensity to delusion formation [38]. Talamo and collaborators report the case of two sisters diagnosed with shared delusions [36]. Both were prescribed antipsychotics and supportive psychotherapy. One committed suicide by overdose of medications, while the other gradually improved during psychiatric hospitalization. Although aggression is reported to be relatively common in folie à deux, no accurate epidemiological data are available that bear this out. 

Capgras and Fregoli syndromes belong to the category of delusional misidentification syndromes [31], classically described but not considered in current diagnostic and statistical manuals for mental disorders. The syndromes can appear in patients with schizophrenia and other related disorders, but they are traditionally associated with DD [31]. The risk of aggression (verbal and physical threats) and dangerousness, in general, has been widely reported in patients with Capgras and Fregoli syndromes [39,40]. These patients generally present with delusions of grandiosity, excitement, and hostility, score high in general psychopathology, and have a previous history of premeditated violence [40]. Specific assessment tools and careful safety measures, as well as the use of psychotropic medication (benzodiazepines and antipsychotics) and frequent clinical monitoring are recommended [41]. 

The Capgras syndrome (or delusions of doubles) is characterised by a belief that an identical clone has replaced a close relative or companion. Acts of violence have been reported, sometimes associated with organic disorders, and positive neuroimaging findings, e.g., right hemisphere abnormalities [42]. Cognitive deficits are associated with a relatively high risk of violence in patients with Capgras Syndrome [42]. The Fregoli syndrome is a delusional belief that one or more familiar people are, in reality, stalkers and persecutors who frequently change their appearance. The association between Fregoli syndrome and aggression is well recognised [43]. Ashraf and collaborators report that the Fregoli syndrome is sometimes associated with the impairment of frontal lobes, particularly the right frontal lobe [43]. No association of frontal lobe lesions with aggression has been found in this syndrome. 

To sum up, patients with DD in older age show a high risk for aggression, especially when persecutory delusions are present. In general, exclusively paranoid symptoms are the ones most frequently associated with aggressivity. The de Clérambault syndrome (erotomania), Othello syndrome (delusion of jealousy), and delusional misidentification syndromes (Capgras and Fregoli) and folie à deux (shared psychosis) are delusional syndromes in which violence and aggressive behaviour are relatively prevalent. 

### 3.3. End-of-life Care in Patients with Delusional Disorder in Old Age 

End-of-life care for patients with delusional disorders has been an understudied topic. As with everyone else, DD patients, as they age, develop serious physical illnesses that require palliative care. Others need care for profound self-neglect secondary to DD symptoms while still others require hospice care at the end of life because of intolerable psychological distress.

Case reports illustrate these issues. Bassirpour and collaborators report the case of a 64-year-old woman with treatment-resistant DD. She had poor insight into her disorder, which was accompanied by an eating disorder that led to progressive loss of weight and severe medical complications [44]. She had dysphagia for solids and liquids and vomited when presented with food. After several failed attempts at treatment with antipsychotics, she was discharged home with the support of the hospice care team. She was found competent to make decisions so could not be fed involuntarily. This is an example of the unsuccessful treatment of DD at the end of life. 

Monden and Zandbergen report the case of a 58-year-old woman with facial basal cell carcinoma present for 11 years [45]. The patient was diagnosed with DD and comorbid personality disorder and refused medical treatment. She was involuntarily admitted to the hospital because of major self-neglect with the risk of death. By that time, the carcinoma was untreatable. 

End-of-life care for people with psychosis has become a focus of interest in recent years because of the demonstrably reduced life expectancy in these populations [46]. A recent systematic review and thematic synthesis explored case studies reporting the organization, provision, and care for people diagnosed with severe mental illness (SMI), which included DD, who were at an end stage of life [46]. In the reported studies, the vast majority of patients were men, and their mean age was 55; psychosis was the most frequent diagnosis and cancer was the most common end-of-life condition. Delayed or late diagnosis and diagnostic overshadowing (the attribution of all symptoms to psychosis) were frequent. Patient denial of illness and lack of insight were complicating factors in end-of-life care in SMI.

Failure to thrive that ends in death is called ‘glissement’ in French medicine, e.g., a slide toward death. It is characterised by confusion, depression, regression, melancholy, high stress, and delusional thinking [47]. Clinicians sometimes decide that the patient “wants to die” and, thus, do nothing. This can be viewed as a dereliction of duty because such delusions demand accurate diagnosis and clinical management [47,48,49].

Decisional capacity is an important issue in the context of palliative care in general. Recently, Kotzé and collaborators carried out a cross-sectional observational study of 100 adults over age 60 who were diagnosed with SMI [50]. Among the assessment instruments were the Mini-Cog and a semi-structured clinical assessment of end-of-life decision-making capacity, which includes the capacity to consent to the proposed treatment. Impaired decision-making capacity was significantly associated with all psychotic conditions (including DD) and with the duration of SMI. While not specifically investigated in DD, decisional capacity in schizophrenia has been shown to improve when the underlying disorder is treated [51]. In schizophrenia spectrum disorders (which includes DD), there is considerable heterogeneity in regard to decisional capacity. Cognitive changes that accompany both age and the underlying illness process make it difficult for the patient to fully comprehend and assess what is happening and what should be done. Medication side effects increase this problem [52]. Pharmacokinetic and pharmacodynamic changes related to aging as well as harmful interactions among multiple drugs increase the risk of toxicity and harmful interactions among therapeutic drugs [53,54]. Surrogate decision-making is often required but difficult to obtain in socially isolated individuals, such as those with DD [55].

Jeste et al. [52] (addressing decisional capacity with respect to consent to research in the context of psychosis) recommend careful repetition and clarification of options to ensure improved understanding.

In DD patients, psychological distress symptoms other than delusions can appear at this time—e.g., depression, anxiety, and delirium [49]. Patients with disabling psychiatric symptoms need attention and care at the end of life that is appropriate for both pre-existing and emerging symptoms. 

Table 1 represents critical situations in patients with DD at the end of life.

There has been recent debate about palliative care approaches (the emphasis on care rather than cure) being a more realistic and humane goal in the context of SMI, irrespective of age and state of physical health [56]. The boundary between curative and palliative approaches to severely ill psychiatric patients can often become blurred. Promoting patient and family input into advance care planning has been advocated [55,57] and more research has been called for in this neglected field. 

## 4. Discussion

As is clear from our findings, DD has not been sufficiently addressed as a diagnostic category distinct from other psychoses. Relatively little is known about its successive stages [58], its associations with accelerated aging on psychotic conditions [59], its comorbidity with structural brain lesions [60], and progressive cognitive deficits [61]. 

The literature that exists suggests that the guidelines for the antipsychotic treatment of middle- and old-age schizophrenia are applicable to delusional disorder, but the fact that DD patients often hold jobs and occupy important family roles means that adverse effects of treatment (sedation, blood pressure effects, parkinsonism) must be scrupulously avoided. Medication doses are, on average, lower than in schizophrenia. Despite relatively good overall function in DD, persecutory delusions or delusions of jealousy can intensify and lead, with age, to agitated and aggressive behaviour. Critical issues are thorough assessment and close monitoring that allows prevention and early intervention should such behaviour occur. Familial and psychosocial approaches are best suited for the initial stages of DD in order to establish a firm therapeutic alliance. Pharmacological initiation is almost always indicated to dampen the force of delusional conviction [62]. Specific antipsychotic treatment of DD has changed in the last several decades. Initial evidence suggested the superiority of pimozide, most specifically in the DD somatic subtype. Subsequent studies found that risperidone and olanzapine were equally effective, and that treatment response was influenced by the presence of cognitive and depressive symptoms, which increase as patients age [2]. More recent studies exploring the effectiveness of pharmacotherapies in the context of DD provide evidence that clozapine and long-acting injectables are more effective than other antipsychotic drugs in terms of reduction of hospitalization rates and work disability [63].

With age, neurological screening and palliative care approaches are often needed. The goal becomes not symptom removal, which may be impossible, but improvement in quality of life. 

## 5. Conclusions

Medical/neurological illness may be the cause for many agitated and aggressive behaviours in patients with DD. This possibility always needs investigation and, if necessary, early treatment. Psychosocial and pharmacological treatments are usually needed to ensure patient, family, co-patient, and staff safety. Although standard treatment of DD traditionally aims to reduce the degree and extent of delusions, with age, safety issues, affective balance, maintenance of adequate function, and quality of life issues become more important than targeting psychotic symptoms. This review underscores the relative lack of research thus far devoted to delusional disorders.

## Figures and Tables

**Table 1:** Patient and physician problem areas in delusional disorders.

Problems in DD Palliative Care		
	Physician	Patient
Clinical Picture	Failure to provide cognitive treatment	Denial of illness Treatment refusal
Decisional Capacity	Failure to explain options Failure to seek surrogate consent	Lack of understanding of options Isolation from family

## Data Availability

The data presented in this review are available upon request from the corresponding author.
